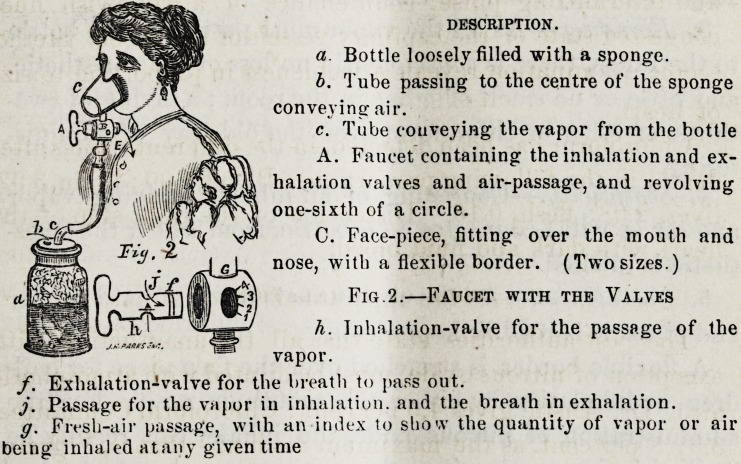# Anæsthetics and Their Administration
*Read before the New York Medical Association, Jan. 15th. 1869.


**Published:** 1869-06

**Authors:** D. H. Goodwillie


### SELECTED ARTICLES.

---

### ARTICLE IX.

### *Anæsthetics and their Administration.**

By D. H. Goodwillie, M.D., D.D.S.

It has been well said that "the discovery of anæsthesia, like many others in science and art, was the result of long-continued and patient effort made by many persons—not by accident, but carried on for years with the definite end in view which was afterwards attained.

"This discovery was thankfully received by suffering hu-

---

*Read before the New York Medical Association, Jan. 15th. 1869.

manity; but by the surgeon, particularly, it received a hearty welcome, as his operations were aided, his usefulness extended and his feelings of humanity spared.

"Although there were shadows that seemed to darken the lustre of this discovery, yet it only appeared to stimulate inquiry, and the result has been a better acquaintance with the agents employed, clearer views of the sources of danger, and more certain rules for safety in their demonstrations."

The Egyptians used many drugs to relieve pain, or assuage grief—to produce intoxication or ecstacy. In the time of the Roman empire, means were employed to mitigate pain in surgical operations.

Pliny and Dioscorides described some medicament for relief of pain. The Chinese surgeons and ancient Scythians inhaled narcotic vapors. The Indians threw tobacco on their camp-fires, to put them in a state of ecstacy.

Even among civilized nations such were used. The priestesses at Delphi became intoxicated by the fumes of narcotic plants before delivering the oracles. May this not have been the germ from which has sprung surgical anæsthesia? Albertus Magnus (thirteenth century) probably knew something of the use of ether as an anæsthetic, for he gives a recipe for its preparation. In the sixteenth century, Porat, of Naples, mentions a soporific medicine.

The anæsthetic effect of nitrous oxide was suggested by Sir Humphrey Davy, in 1776, as seen by the following extract from his work: "As nitrous oxide in its extensive operations appears capable of destroying physical pain, it may probably be used with advantage during surgical operations in which no great effusion of blood takes place." This idea of Davy's found a practical demonstrator in the person of Dr. Horace Wells, to whom, without doubt, belongs that honor. The successful trial of surgical anæsthesia occurred several years before Jackson proposed, or Morton used ether. That the brain of Horace Wells was the modern scource and origin of anæsthesia has been fully established.

An anæsthetic is a substance capable of abolishing the function of sensation. True anæsthetics are local anæsthetics, because capable of depriving it of sensation only. In general anæsthetics there is loss of motion, perception, thought, conciousness, etc.

*List of Anœsthetics.*

| NAME. | SYMBOL. | PROPERTIES. | Boiling point. Fahr. | Gas or vapor. Density H=i. |
|---|---|---|---|---|
| Nitrous oxide gas.... | $NO$. | Supports combustion.... | ...... | 22 |
| Carbonic oxide gas.... | $CO$. | Burns in oxygen.... | ...... | 14 |
| Carbonic acid gas.... | $CO_2$ | Prevents combustion.... | ...... | 22 |
| Light carburetted hydrogen gas.. / Hydrate of methyl or marsh gas.. | $CH_4$. | Burns in air.... | ...... | .8 |
| Methylic alcohol.... | $CH_4O$ | Vapor burns in air.... | 151 | 16 |
| Methylic ether gas.... | $(CH_3)_2O$ | Burns in air.... | ...... | 23 |
| Chloride of methyl gas.... | $CH_3Cl$ | ....do.... | ...... | 25.25 |
| Bichloride of methylene.... | $CH_2Cl_2$ | ....do.... | 88 | 42.5 |
| Terchloride of formyl (chloroform) | $C_2HCl_3$ | Vapor extinguishes flame | 142 | 59.75 |
| Tetrachloride of carbon.... | $CCl_4$ | ....do.... | 172 | 77 |
| Heavy carburetted hydrogen gas / Olefiant gas or ethylene.... | $C_2H_4$ | Burns in air.... | ...... | 14 |
| Ethylic alcohol (absolute alcohol).. | $C_2H_6O$ | Vapor burns in air.... | 172 | 23 |
| Ethylic ether (oxide of ethyl, sulphuric ether).... | $C_4H_5O$ | ....do.... | 96 | |
| Chloride of ethyl.... | $C_2H_5Cl$ | ....do.... | 52 | 32.25 |
| Bichloride of ethylene (Dutch liquid) | $C_2H_4Cl_2$ | ....do.... | 175 | 49.5 |
| Ametic alcohol (fusil oil).... | $C_5H_{12}O$ | ....dd.... | 270 | 44 |
| Hydride of amyl.... | $C_5H_{12}$ | ....do.... | 86 | 36 |
| Amylene.... | $C_5H_{10}$ | ....do.... | 90 | 35 |
| Hydrate of caproyl (light pet. spirit) | $C_6H_{14}$ | ....do.... | 154 | 43 |
| Benzol.... | $C_6H_6$ | ....do.... | 180 | 39 |
| Turpentine spirit.... | $C_{10}H_{16}$ | .... | 320 | 68 |

### Compound Anæsthetics.

*Chloride of methyl in ether—Chloride of methyl in chloroform.*

Among the many anæsthetics, I propose to take up only those in general use, viz.: chloroform, ether, bichloride of methylene, and tetrachloride of carbon.

### CHEMISTRY OF CHLORFORM.

It is a colorless liquid. Aromatic and penetrating smell—taste sweetish—sp. gr. 1.491, chem. formula $C_2$ $HCl_2$ or 12 parts carbon, 1 part hydrogen, 105 parts chlorine. It is made by the distillation of rectified spirits with water and chloride of lime—the addition of quicklime renders the product more abundant and pure. It should be purified by washing with water, then shaken with a solution of carbonate of soda, and then redistilled.

The adulterations of chloroform are:

I. Alcohol or ether, which reduces its strength.

II. Methyl compounds.

III. Product of decomposition.

A ready test for alcohol is to drop some chloroform in distilled water. If pure, the globules of chloroform will sink to the bottom and preserve their transparency; if alcohol be present, they will have a milky appearance. The test for ether is the same as alcohol, by the smell.

Test for methyl compounds is strong sulphuric acid; becomes black when mixed with chloroform. Time, air, and light, produce a variety of changes in chloroform by the formation of certain hydrochloro-carbons, hydrochloric acid and free chlorine. Free chlorine may be detected by adding a little chloroform to distilled water, and then add a little solution of nitrate of silver; if chlorine is present, a white precipitate will be formed.

Chloroform evaporates at all temperatures. Air at 40° Fahr. can retain 6 per cent. of chloroform vapor. Air at 60° Fahr. 12 per cent. This fact ought not to be forgotten in the administration.

### CHEMISTRY OF ETHER.

Ether, chemical formula $C_4 H_5 O$, or by weight, 24 parts carbon, 5 parts hydrogen, and 8 parts oxygen.

Prepared by distilling equal parts of alcohol and sulphuric acid. A colorless and limpid liquid, of peculiar odor and hot taste, specific gravity, at 60° Fahr., .720, boils at 96° Fahr. With oxygen or atmospheric air it forms an explosive mixture, and kept in contact with air, produces acetic acid. Dissolves in alcohol in all proportions, and is often diluted with it; purified by water, which unites with it. Chloroform compared with ether is much more powerful.

Dr. E. R. Squibb gives the following test for ether: Half fluid ounce of the stronger ether, evaporated spontaneously from a breakfast-plate, should give, as the last portions pass off, only a faint aromatic odor of light oil of wine, and should leave an odorless, tasteless residue upon the surface of the plate. When shaken in a test tube with an equal volume of water, it should lose 10 per cent. of its volume. In a test-tube half filled and grasped in the hand for a short time, it should boil actively upon the addition of small fragments of broken glass.

CHARACTER OF BICHLORIDE OF METHYLENE. ($CH_2 Cl_2$)

(By weight, C. 6 parts, $H_2Cl.$, 70.)

1. It is an effective general anæsthetic, producing as deep insensibility as chloroform.

2. In action it is rather more rapid than chloroform, but, to develop effects more of it is required, in the proportion of six parts to four.

3. It produces in the second degree, less prolonged narcotism than other anæsthetics.

4. When effects are developed fully, the narcotism is very prolonged.

5. Its influence on the nervous centres is uniform, and it creates little, if any disturbance, or break of action between the respiration and circulating functions.

6. Its final escape from the organism is rapid; symptoms of recovery are sudden.

7. In some cases it produces vomiting.

8. When it kills, it destroys by equally paralyzing the respiring and circulating functions.

9. It interferes less with the muscular irritability than perhaps any other anæsthetic.

10. It combines with ether and chloroform in all proportions.

I have used this agent several times, and find that it accords with the above character, and trust it may always prove so.

It will be seen that the only difference between the bichloride of methylene and chloroform is, that it has one atom more of hydrogen, and one less of chlorine and carbon.

TETRACHLORIDE OF CARBON. ($CCl_4$)

(By weight, 6 parts, C. and 140 Cl.)

It has a pleasant odor, somewhat resembling that of quinces.

Anæsthesia is rapidly produced by it, and easily sustained with or without entire loss of consciousness, and the effects pass off very quickly.

Not usually any excitement or struggling before anæsthesia supervenes, or followed by any sickness.

It possesses a great point of interest in immediately allaying pain from any cause, headache, dysmenorrhœa, suffering, etc. It is said to be valuable in inducing quiet and refreshing sleep.

In midwifery it removes pain without necessarily destroying consciousness, or interfering apparently with the expulsive efforts of labor.

Having had but little experience with this agent as an anæsthetic, I cannot therefore pass judgement on it. I am not, however, thus far very strongly biassed in its favor.

The effects of inhalation of these anæsthetics are both local and general. On a sensitive surface it acts as a caustic. The irritation of the air-passages is due to this fact; thus we have the spasm manifested by coughing, etc. When the narcotic effect is produced, the spasm is controlled. Irritation is changed for partial paralysis.

The physiological action of these anesthetics is to produce I. A stimulus—an excitation of the functions. II. A suppression—a retardation of the functions. Small quantities, diluted with air, prolong the former—large quantities suddenly collapse the functions. The power over muscular motion is unequal, but abrogation of sensation is common to all. The mental processes are less affected by nitrous oxide.

Circumstances modify the action these agents may have upon the system. Diffusibility, volatility, solubility, all influence their effects.

While water can hold in solution one-ninth of its weight of ether, it dissolves only $\frac{1}{280}$ of chloroform.

We see from this that the quantity *inhaled* is *not* the quantity *absorbed.* All these agents act by absorption into the blood, whether taken into the lungs or stomach, or injected under the skin. Here, let me remark, is opened up a wide field of research. Gases ( N.O., for instance) are readily absorbed and easily eliminated. Vapors are much less so. Elimination is accomplished in less time, if the anæsthetic agent is taken into the lungs, than in any other way, as the blood is not so heavily loaded.

When a quantity of vapor or gas is breathed, it is brought into contact with six hundred millions of air cells of the human lungs, the superficial extent of which, as estimated by Linderman, is not less than 2,642 square feet. In inducing anæsthesia, fifteen or 20 cubic inches of the anæsthetic mixture, fifty or sixty times over, are brought into contact with this surface, and manifest their effects by direct action on the central parts of the nervous system, through the medium of the blood; or they may act on the blood, modifying that interchange of elements necessary to perfect health; or in other words, that narcosis is suspension of oxygenation.

ACTION OF ANÆSTHETICS ON THE BLOOD.

Ether gives the blood a dark-blue color, prevents rearterialization, and dissolves the blood-corpuscles setting free the hematin.

Chloroform turns the blood a brilliant scarlet color; does not dissolve the blood-corpuscles, as ether, but destroys a greater number of them, setting free the hematin and crystallizing it.

Dr. Geo. Harley says: "The common property of all narcotics and anæsthetics is to diminish the energy of interchange between the constituents of the air on the one hand and the blood on the other."

Causes of difference in color of blood are due less to change of composition than to change of form. Distending the corpuscles darkens the blood, making them more convex; when concave, it brightens the blood.

The first general effects on the organism, of the anæsthetics, when inhaled, are stimulant. As soon as the narcotic begins, the senses become affected; and, about the time sensation is lost, there occurs a muscular tremor. This indicates the severance from the central nervous power; the muscles, which are now left to their own individual influence—heat, electricity, etc.—begin to relax for a want of coördinating power. Coördination, being a compound of sensation and motion, is perfect only when the nerve-fibres are in equal degree capable of transmitting impressions from the centres.

When muscular tremor subsides, the patient is in a state of complete insensibility, having the appearance of sleep: the pupil of the eye somewhat contracted; pulse beating rather slow; the breathing regular; begins to snore a little.

There is now a very delicate balance in the organism, in this state of induced anæsthesia. Dr. Sansom says of it: "On the one side, is Being robbed of many of its attributes; on the other side, is death. Compensating oxygenation maintains the one, while insufficient oxygenation induces the other."

Dr. Sansom divides narcotism into three stages, viz.: 1. Perversion of consciousness; 2. Abolition of consciousness; Muscular relaxation. Or, in other words: 1. Sopor; 2. Stupor; 3. Stertor.

Narcotism is suspended oxygenation. Whatever produces, to a certain extent, insufficient aeration of the blood, produces narcosis, and *vice versa.*

To produce anæsthesia the following causes combine, viz: 1. Preventing oxygenation; 2. Diminishing arterial supply; 3. Sluggishness or flow in the capillaries; 4. Subdual of energy of the nerve-filaments distributed to the lungs; 5. Shallowness of respiration, contributing to prevent free entrance of air into the blood.

I give here the views of Dr. Anstie, as the most rational, and supported by facts, as to the mode of death by chloroform:

"I believe death to take place in two ways, depending on the rapidity of absorption of the vapor. When the impregnation of the blood takes place with moderate rapidity, the sympathetic nervous system is the ultimum moriens and death begins at the lungs. When, on the contrary, the circulation becomes very rapidly charged with a large proportion of chloroform, the narcotic effect may fall with such force upon the sympathetic nerves as to extinguish their vitality at once.

"From the fact that, in an immense majority of reported fatal cases, the symptom of danger was confessedly the failure of the pulse and the blanching of the countenance, the conclusion appears strongly indicated that paralysis of the heart is *the* source of danger in surgical chloroform narcosis."

Death by shock is spoken of by a writer of an article in the *American Journal of Medical Sciences:*

"The fatal impression being a sudden influence upon the branches of the par-vagum in the lungs, produced by the inhalation of undiluted vapor of chloroform during the administration. That sudden impressions on the peripheral extremities of nerves produce a profound effect upon the internal vital organs, is one of the best known facts of physiology."

The report of the Committee of the Medical and Chirurgical Society of London contains the following:

*Stage of Anæsthesia at which Death occurs.*

| | |
|---|---|
| Before full effects of chloroform, | 50 |
| During " " " | 52 |
| Not stated | 7 |
| Total | 109 |

Thus it will be seen that a great many deaths occurred before insensibility was established, and few when a profound action of the anæsthetics was sustained.

It is a generally-acepted fact that ether is less dangerous than chloroform, but testimony, by truthful authors, report deaths from ether. Trousseau reports nineteen. The Boston Society for Medical Improvement reports thirty-six.

Chloroform death-rate is much above that of ether in frequency. The relative danger of these two agents is impossible without figures, which cannot be obtained. Death occurs chiefly among males.

Dr. Snow's proportions are three males to two females; Kidd's, four to one; Sansom's, 2.8 to one. As to age, thirty years is stated as the average. The strong and muscular resist anæsthesia most, and, when produced, it is often deep coma and profound stertor. The feeble bear anæsthetics better than the strong. Children are the best subjects of all. Women are better than men. Many deaths occur in the inebriate. Fatty degeneration of the heart is to be feared more than any other disease. The following is the diagnosis: Tendency to fainting; occasional dyspnœa, from congestion of the lungs; indication of atheroma of the arteries; feeble and intermitting pulse; countenance of a yellowish hue; congested state of the capillary vessels of the cheek. Stethoscopic examination reveals a feebleness in proportion to size of heart.

Chloroform has been detected, in the different tissues after death, in the following proportions: Blood, 1.00; brain, 3.92; liver, 2.08; flesh, 0.16. Distention of the right side of the heart with dark and fluid blood.

MODE OF INHALATION.

The best authorities state that all the anæsthetics ( with exception of nitrous oxide) must be diluted with atmospheric air. Dr. Anstie gives 4.5 per cent.; Chloroform Committee, 3.5; 5 per cent. as the maximum amount—that is, 5 grains of chloroform to 100 cubic inches of air, The same may be said of bichloride of methylene and tetrachloride of carbon.

Ether being a much less powerful agent, can be used in a much larger per cent.

There are various modes used to administer the anæsthetics (vapor not gases,) such as the handkerchief, towel, sponge, cone, and by inhalers.

The great objection to nearly all these modes is, that we are in ignorance as to the strength of the vapor inhaled at any given time. At one moment, the air may be strongly impregnated with vapor, and, at another, pure atmospheric air is inhaled, so that there is no certainty about it.

The local effect on the air passages, when *strong* vapor is inhaled, are to produce spasms, manifested by coughing, and, if continued, struggling and possibly sickness. On the other hand, if the vapor is well diluted with atmospheric air at first, the air passages come gradually under the influence of the vapor, and the patient passes calmly into the anæsthetic sleep.

To accomplish this desireable end, I have constructed an inhaling apparatus, which will be readily understood by a reference to the accompanying figure:

DESCRIPTION.

*a.* Bottle loosely filled with a sponge.

*b.* Tube passing to the centre of the sponge conveying air.

*c.* Tube conveying the vapor from the bottle

A. Faucet containing the inhalation and exhalation valves and air-passage, and revolving one-sixth of a circle.

C. Face-piece, fitting over the mouth and nose, with a flexible border. (Two sizes.)

FIG. 2.—FAUCET WITH THE VALVES

*h.* Inhalation-valve for the passage of the vapor.

*f.* Exhalation-valve for the breath to pass out.

*j.* Passage for the vapor in inhalation, and the breath in exhalation.

*g.* Fresh-air passage, with an index to show the quantity of vapor or air being inhaled at any given time

The relative proportions of vapor and air are changed by the revolution of the faucet (*A*) over the inhalation-valve (*h.* Fig. 2) and the air passage (*G*, Fig. 2.) These proportions shown by the index at G. Thus revolving from 1 to 4 increases the vapor, and in the same proportion decreases the air from 4 to 1 *vice versa.* 1 is the minimum amount of vapor and the maximum amount of air, 4 the maximum amount of vapor and minimum of air.

When the patient inhales, the air passes into the bottle at *b*, causing the liquid to evaporize on the upper half of the sponge, and passing out at *c* into the inhaler, to be inhaled, the amount of vapor or air being regulated by the revolution of the faucet over the inhalation-valves and air-passage.

The bottle should never be more than half full, so as to allow evaporation on the upper half of the sponge.

The value of this inhaling apparatus consists in—

1. *Its safety.*—Air is at all times inhaled with the vapor, producing *good* anæsthesia without asphyxia. It is under complete control of the anæsthetist.

2. *Efficiency.*—The gradual inhalation of the vapor produces *less spasm* of the *epiglottis* (coughing) *struggling* or *sickness*, Anæsthesia is quietly produced and maintained. Rapid recovery from the anæsthetic.

3. *Economy.*—As all the vapor must past from the bottle to the lungs, there is consequently no loss of the anæsthetic. and little or no smell of ether in the room ; a saving of two-thirds of ether or chloroform over the old way of administering.

4. *Simplicity.*—Consisting of an inhaler (mixing vapor and air at will,) connected to a bottle, from which the anæsthetic is inhaled.

5. *Cleanliness.*—As the apparatus is made of hard rubber and glass, it is readily kept clean.

A flexible border is stretched over the face-piece for children, or inhalation by either the mouth or nose. For the administration of nitrous oxide, the inhaler can readily be applied by connecting it to the gasometer or bag instead of the bottle.

I present here a summary, from a record of cases of anæsthesia with this apparatus, from the report of Bellevue and Charity Hospitals.

Whole number of cases, 50 ; males, 37 ; females, 13. Maximum age of patient, 58 years; minimum age of patient, 15 months.

Maximum time of ether inhalation, 1h. 10 minutes.
Minimum " " " 8 minutes.
Maximum time to produce anæsthesia, 12 minutes.
Minimum " " " 1½ minutes.
Whole amount of ether used, 256 ℥.
Minimum " " " 2⅔ ʒ.
Maximum " " " 6½ ℥.
Average amount of ether used, 2 ℥ 5 ʒ.
Average time to produce anæsthesia, 5 minutes 7 seconds.
Average time anæsthesia was kept up, 27 minutes 24 seconds.
Average age of patient, 28½ years.

Or, on an average of the above cases, 2 ℥ 5 ʒ of ether will produce anæsthesia 5.1 minutes, and maintain it for 27 minutes 24 seconds, in a patient aged 28½ years.

Only four cases of sickness which possibly might have been prevented somewhat by previous care. One, a case of amputation in a man with delerium tremens, died on sixth day. Autopsy showed an abscess of the brain, fatty degeneration of the heart with old pericarditis. He went through the etherization very well. Had no delirium after the opertaion.

*An impromptu inhaler* may be made as follows: Procure an oblong piece of stiff paper ( a news paper will do,) about eighteen inches long by twelve inches wide. Place upon it a napkin or pocket handkerchief and fasten it by a few pins. Now roll the paper so as to form a hollow cylinder of about four inches in diameter, the inside being lined by the napkin to receive the anæsthetic. Air will enter the hollow of the cylinder, evaporating the anæsthetic and at the same time diluting it.

Mixtures of chloroform, ether, and alcohol have been advised, to avoid danger from the depressing influence on the heart's action.

The unequal rate of evaporating of the fluids is a prominent objection among others.

The best dilution to my mind, is a *well-measured sufficiency of atmospheric air.*

The following general rules are to be observed in administering the anæsthetics:

I. The anæsthetist should confine himself *exclusively* to the administration, and watch closely the symptoms.

II. The indices of danger are—1, the respiration ; 2, the pulse ; and 3, the countenance.

III. To prevent sickness, do not allow the patient any solid food *four hours* or liquid nourishment *two hours* before the operation. All alcoholic stimulants to be given at least *one half hour* before the anæsthetics are given, as this should be through the system and not in the stomach as one cause of sickness.

IV. Reassure the patient by gentle words and actions. Place your patient in a horizontal position, with the head raised a little, to let mucus and saliva go down the throat, and facilitate the breathing.

V. Loose the clothes and give all the muscles of respiration free play— especially the diaphram. Keep the body warm, and thus assist circulation and respiration.

VI. Avoid the spasm of the epiglottis (manifested by coughing,) by the gradual inhalation of the vapor, as this spasm undoubtedly affects the stomach also.

VII. Muscular tremor is always followed by muscular relaxation. In the strong and healthy this change is often quite sudden, so that care must be exercised, when the tremor subsides, to let the patient breath fresh air freely.

VIII. Cover the patient's eyes with a napkin while recovering from the anæsthetic, until sensibility and consciousness fully return, as it keeps them quiet (and they not unfrequently fall into a natural sleep,) for applying dressings, etc.

IX. Fresh air is the best stimulant for recovery. Let the patient exhale freely, to eliminate the vapor from the system. Give a warm cup of coffee or tea, and, if the patient is weak, add a little wine or brandy.

*Resuscitation*, in cases of danger, is accomplished by the only efficient stimulus, respiration, either natural or artificial.

Draw forward the tongue with your fingers or forceps, and keep it there by some means. Use either mouth-to-mouth inflations or by the methods of resuscitation by Dr. Marshall Hall, Sylvester, or Howard. The first is probably well known, and for that reason, and also that I much prefer the others, I will not give it here.

Dr. Sylvester's method is as follows: Draw the tongue forward. Place the patient on a flat surface on his back, slightly inclined from his feet upward, the chest being elevated on an impromptu cushion. Then grasp the arms and press them firmly against the sides of the chest. Next, immediately to raise the arms by the sides of the head and keep them stretched steadily upwards and forward for two seconds. Repeat these measures alternately, with long perseverence and unfailing regularity, fifteen times in a minute.

Dr. Benjamin Howard's* *Direct Method* of artificial respiration is as follows:

"The patient is laid on the ground upon his back, his arms fully extended backward and outward, a firm roll of clothing being placed beneath the false ribs, so as to throw their anterior margin prominently forward. The tongue being held forward by an assistant the operator facing the patient, kneels astride his abdomen, and places both hands so as the balls of the thumbs rest upon the anterior margins of the false ribs, the four fingers falling naturally into four of the lower corresponding intercostal spaces on each side.

"The elbows of the operator being then planted against his sides, he has but to throw himself forward, using his knees as a pivot, and the entire weight of his trunk is brought to bear upon the patient's false ribs. If at the same time, the fingers of the operator grasp and squeeze the false ribs toward each other, these combined actions crowd the false ribe upward and inward, producing the greatest possible motion of the diaphragm and displacement of the contents of the pulmonary air-cells.

"The operator then suddenly lets go and returns to the erect position upon his knees, where both the in-rush of air and the natural elasticity of the ribs at this part cause instant return to their normal position.

"This repeated with proper rythm and frequency, constitutes the entire process.

"The advantages of this direct method over the indirect method of Sylvester and Hall, Dr. H. claims are:

"1. It is more simple.

"2. The degree of compression is felt and can be regulated by the operator.

"3. All the available anatomical Means for the displacement of air in the cavity of the chest are completely used.

"4. While the necessary motions are in progress, the tongue may be steadily held out, the limbs and entire body be dried and rubbed without interfering with the operator.

"5. No time is lost in superfluous motions.

"6. It is less fatiguing to the operator.

"7. It is more quickly taught to a bystander."

Galvanism of the phrenic nerves may also be used by placing one of the poles (armed with a wet cloth or sponge) over the situation of the phrenic nerve where omo-hyoid and sterno-mastoid cross the other pole along the course of phrenic, or at its periphery over the diaphragm (seventh intercostal space.) The current should be interrupted at regular intervals, to imitate the natural respiration.

---

**Medical Record* December 15, 1868, p. 467.

This can be applied in Dr. Howard's method, without interruption. Warmth and friction should always be applied. Warm air to the lungs by a bellows, when possible. When air cannot reach the lungs by the mouth, tracheotomy may be performed. Stimulants may be given by the rectum and by the mouth when the patient has recovered so as to swallow.—*New York Medical Journal.*

## Figures and Tables

**Fig.2. f1:**